# Endomyometriosis of the Rectum With Disseminated Peritoneal Leiomyomatosis 8 Years After Laparoscopic Myomectomy: A Case Report

**DOI:** 10.3389/fsurg.2021.666147

**Published:** 2021-04-16

**Authors:** Giorgio La Greca, Cristina Colarossi, Paolo Di Mattia, Cecilia Gozzo, Marco De Zuanni, Eliana Piombino, Lorenzo Memeo

**Affiliations:** ^1^Surgical Oncology Unit, Department of Experimental Oncology, Mediterranean Institute of Oncology, Catania, Italy; ^2^Pathology Unit, Department of Experimental Oncology, Mediterranean Institute of Oncology, Catania, Italy; ^3^Radiodiagnostic and Radiotherapy Unit, Department of Medical and Surgical Sciences and Advanced Technologies “GF Ingrassia”, University of Catania, Catania, Italy

**Keywords:** endometriosis, endomyometriosis, uterus-like mass, leyomiomatosis, myomectomy

## Abstract

Endomyometriosis is a rare finding and it can be challenging to diagnose and to treat. It can arise in the uterus, in the ovary, in the broad ligament, in the peritoneal surface and in other pelvic structures. Usually patients with endomyometriosis are asymptomatic, but symptoms could occur due to large dimensions or site of the mass. We present a case of a 49-year-old woman with a symptomatic pelvic mass in the rectal wall, with no history of endometriosis, who underwent laparoscopic myomectomy 8 years earlier.

## Introduction

Endomyometriosis is a benign and relatively rare disease first reported by Cozzutto et al. in the ovary in 1981 (1). The term endomyometriosis was first used by Rohlfing et al. (2) to define a lesion composed by mucosa resembling the endometrial gland and stroma, surrounded by often concentric myometrial-like smooth muscle. The lesion usually is located in the adnexal region (3, 4) but was found also in the gastrointestinal tract (4) or the liver (5).

Here we report a case of a 49-year-old woman with a symptomatic pelvic mass in the rectal wall, with no history of endometriosis, who underwent laparoscopic myomectomy 8 years earlier.

## Case Study

**Figure 1:**
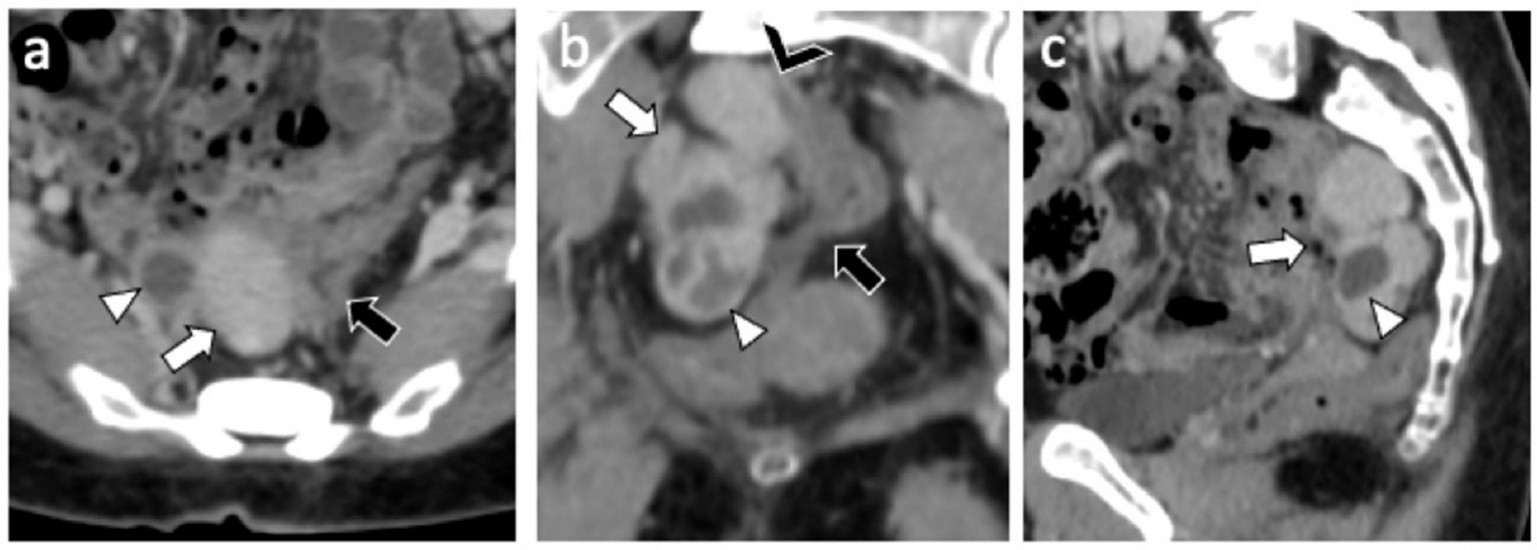
CE-CT exam: Axial **(a)** coronal **(b)** and sagittal **(c)** CE-CT images shows a 6 cm × 4 cm multilobed mass (white arrow) characterized by both solid (Hyperdense) and cystic (hypodense) component (white arrowhead) with homogeneous enhancement and close proximity (black arrowhead) to sigmoid wall (black arrow).

**Figure 2:**
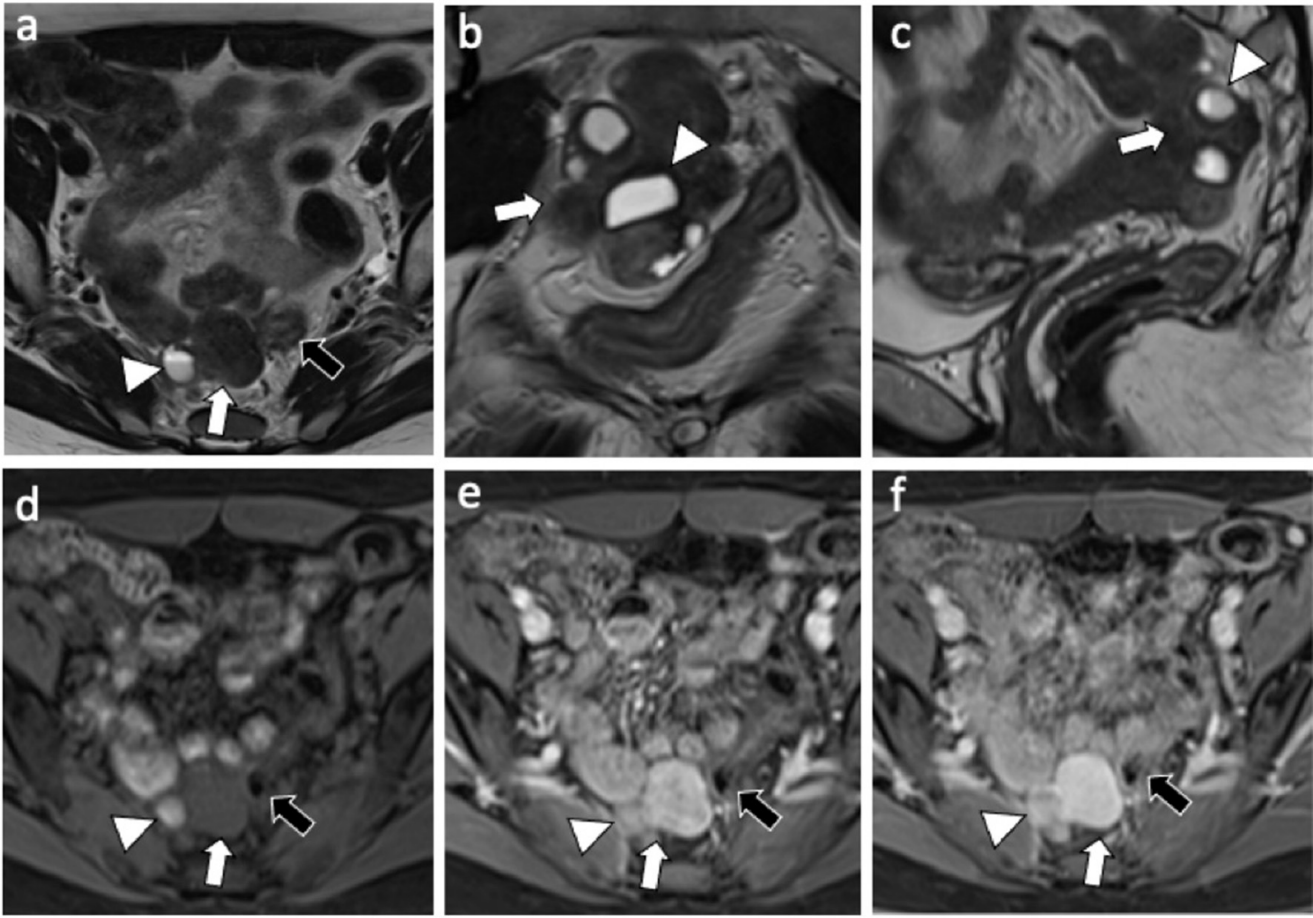
MRI exam: Axial **(a)** coronal **(b)** and sagittal **(c)** T2-weighted images shows a 6 cm × 4 cm multilobed mass (white arrow) characterized by both solid and cystic component (white arrowhead) with fluid-fluid level (hemorragic); axial T1-weighted fat-suppressed pre-contrast **(d)**, and arterial **(e)** and delayed **(f)** post-contrast phase shows omogeneous enhancement of the mass with no clivage plane toward the sigmoid wall (black arrow).

A 49-year-old nulliparous patient with history of hypertension and chronic otitis media came to our attention complaining 3 months of pelvic pain. She had undergone a laparoscopic myomectomy 8 years earlier. Routine laboratory tests were normal and a transvaginal ultrasound revealed a normoverted uterus, with normal size, typical signs of adenomyosis and showed a large retro-uterine mass measuring 59 × 31 × 42 mm with an increased vascularity. A contrast-enhanced computed tomography (CE-CT) scan of abdomen and pelvis showed, in the pre-sacral space, the presence of a multi-lobed mass (6 cm × 4.5 cm in diameters) characterized by both solid and cystic components with homogeneous enhancement in close proximity to rectal wall (Figure 1). A contrast-enhanced magnetic resonance imaging (CE-MRI) of the pelvis confirmed the well-defined, multi-lobed mass characterized by solid and cystic component with hemorrhagic content (fluid-fluid level); homogeneous enhancement of the mass and adherence to the rectal wall were detected (Figure 2).

An endoanal ultrasound suspected a tight connection of the mass to the rectum, therefore a colonoscopy was performed to investigate the possible intestinal origin of the tumor but it excluded any visible intraluminal lesions.

An abdominal laparoscopic examination was then performed. It showed normal uterus and ovaries and the presence of a trilobed mass adherent to the anterior wall of the rectum and two 2 cm nodules in the omentum and in the abdominal wall, the latter in the site of the previous left port.

In addition, pigmented endometriotic nodules were found in the pouch of Douglas and in the lateral abdominal wall.

Since it was not possible to exclude the malignant nature of the rectal trilobed lesion, a laparoscopic low anterior resection of the rectum (LAR) with an end-to-end colorectal anastomosis without lateral ileostomy was performed. A partial omentectomy to remove the abdominal masses was also performed.

**Figure 3:**
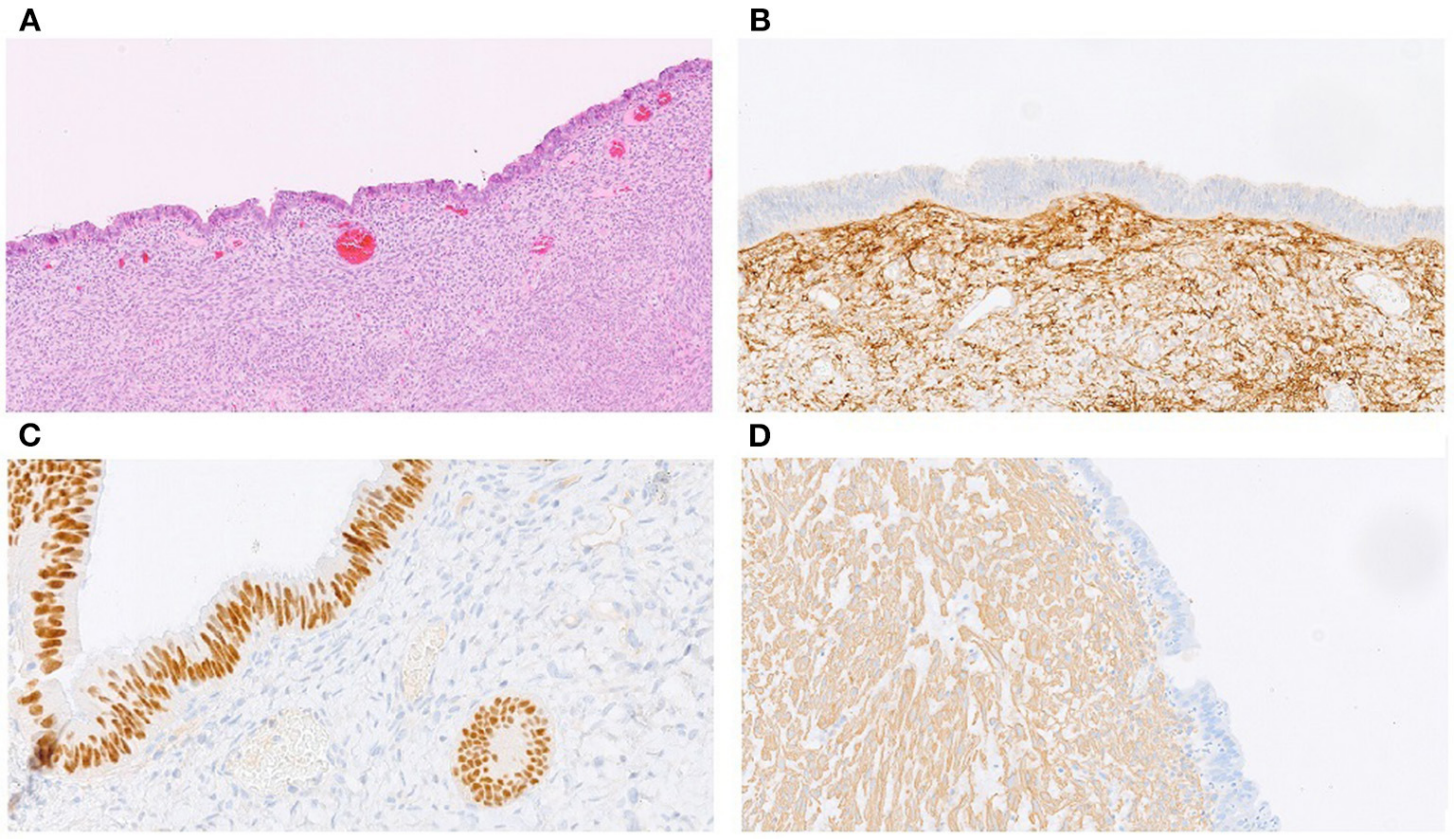
Pathological findings: **(A)** H&E staining of the mass, showing a thick smooth muscle layer lined by endometrial epithelium and stroma (10x); **(B)** immunohistochemical analysis of CD10 showing a diffuse expression in the endometrial stroma without staining of the endometrial mucosa (20x); **(C)** PAX8 nuclear expression limited to the endometrial mucosa (40x); **(D)** smooth muscle actin (SMA) expression in the smooth muscle layer (10x).

Histological examination showed that the trilobate mass was composed of a thick muscular layer surrounding a discrete glandular epithelium. No sign of mitotic activity was seen in the myometrium or the glandular or stromal component of the mucosa. Immunohistochemistry demonstrated a diffuse nuclear progesterone and estrogen receptor expression in the glands, stroma, and muscle bundles, a diffuse nuclear expression of PAX8 in the glandular component of the mucosa and a diffuse expression of CD10 in the stromal component. The muscular component was diffusely positive for smooth muscle actin (Figure 3).

The two 2 cm omental and abdominal wall nodules were both leiomyomas with no evidence of glandular component or mitotic activity.

Post-operative days were uneventful and the drain was removed on third post-operative day and the patient was discharged on 6th day after surgery.

A digital rectal examination and a CT scan of abdomen and pelvis, performed 6 months from surgery, have excluded a local recurrence and so far, almost 1 year post surgery, the patient doesn't refer symptoms.

## Discussion

We described a very unusual lesion. According to the literature, few cases of endomyometriosis have been reported so far (6). In addition, the association between leyomiomatosis peritonealis, endometriosis and endomyometriosis has been only rarely described (7).

The mechanism underlining the presence of endometrial and stromal tissues together with smooth muscle is still not clear. It has been proposed that sub-mesothelial multipotential stem cells can differentiate in smooth muscle cells, endometrial glands and stroma. The subperitoneal mesothelium is often defined “the secondary mullerian system.” During embryogenesis, the distal segments of the two müllerian ducts, which represent the “primary müllerian system,” fuse to form uterus, cervix, and proximal third of the vagina. The proximal müllerian ducts remain separated to become the fallopian tubes. Remnants of the most proximal müllerian ducts, that do not participate in organogenesis, constitute the secondary müllerian system. This system maintains its ability to differentiate into specialized epithelia or stroma into adult life (8). The origin from mullerian system is a very complex mechanism and represents a crucial theory of the pathogenesis of endometriosis (9).

Endometriosis is defined as the presence of endometrial tissue outside the endometrium. It usually consists of endometrial glands and stroma, however, smooth muscle metaplasia has been observed in various endometriotic foci. It has been showed, recently, that peritoneal endometriosis develops smooth muscle proliferation with expression, in addition to common markers of smooth muscle differentiation, of oxytocin and vasopressin receptor like in myometrial cells (10).

Extensive amounts of smooth muscle in endometriosis have been classified as endomyometriosis (11). Cozzutto in 1981 (1), and Rosai in 1982 (12), called this lesion “uterus-like masses,” because of the presence of a central cavity lined by endometrial tissue and myometrium resembling a uterus. According to the literature, the terms endomiometriosis and uterus-like mass indicate the same morphologic features. The frequent locations of endomyometriosis are the ovary (3), uterus (13), and broad ligament (14). Less often is observed in abdominal cavity (15).

In our case the patient had a lobular peri-rectal mass with cystic aspect, a well-organized endometrium and a smooth muscular layer with irregularly arranged muscle fibers histologically identical to myometrium. In addition, two omental leiomyomas were classified as leiomyomatosis peritonealis disseminated and two pigmented endometrioid lesions were seen in the pelvis. We could speculate that all those processes are correlated to mullerian epithelium metaplasia, however, our patient had a medical history of laparoscopic myomectomy in 2012.

Al-Talib and Tulandi (16) proposed an iatrogenic mechanism, due to the possible peritoneal implant of fragments during laparoscopic surgery. In a recent review of the literature (17) 11 out of 13 patients affected by peritoneal leiomiomatosis had a history of laparoscopic surgery for uterine myoma. Miyake et al. (18) performed molecular genetic analysis of a case of leiomiomatosis peritonealis disseminated (LPD) arising 2 and 6 years after the initial surgery for uterine leiomyoma. They demonstrated that the nodules were genetically related since all of them showed X-chromosome inactivation pattern an identical pattern of loss of heterozygosity (LOH) so they suggested a clonal origin of these fibroids. Moreover, some of the nodules were located in proximity of laparoscopic port site. The etiology of endomyometriosis and LPD is still unclear and various factors must be considered in the pathogenesis. Accumulating evidence suggests that both iatrogenic factors and mullerian etiology are important causes.

On a clinical point of view, rarely these lesions are asymptomatic incidental findings and most frequent symptoms are represented by dyspareunia, dysuria and menstrual constipation. These symptoms are often clinically unrecognized and patients are often treated for pelvic inflammatory disease (8).

Endomyometriosis is a very rare disease and should be taken in account in patients with a previous history of myomectomy, and particular care is necessary during laparoscopic extractions of myomas.

## Data Availability Statement

The original contributions presented in the study are included in the article/supplementary material, further inquiries can be directed to the corresponding author.

## Ethics Statement

Ethical review and approval was not required for the study on human participants in accordance with the local legislation and institutional requirements. The patients/participants provided their written informed consent to participate in this study.

## Author Contributions

GL, PD, CG, EP, and MD drafted the manuscript. CC and LM edited the manuscript. All authors were involved in the clinical care of the patient and approved the final version of the manuscript at time of submission.

## Conflict of Interest

The authors declare that the research was conducted in the absence of any commercial or financial relationships that could be construed as a potential conflict of interest.
